# The Use of Noninvasive Velacur® for Discriminating between Volunteers and Patients with Chronic Liver Disease: A Feasibility Study

**DOI:** 10.1155/2024/8877130

**Published:** 2024-01-17

**Authors:** Michael P. Curry, Edward Tam, Caitlin Schneider, Noha Abdelgelil, Tarek Hassanien, Nezam H. Afdhal

**Affiliations:** ^1^Division of Gastroenterology, Hepatology and Clinical Nutrition, Beth Israel Deaconess Medical Center, Boston, MA, USA; ^2^Pacific Gastroenterology Associates, Vancouver, BC, Canada; ^3^Sonic Incytes Medical Corp., Vancouver, BC, Canada; ^4^Southern California Research Center, Coronado, CA, USA

## Abstract

**Background and Aims:**

Nonalcoholic fatty liver disease is the leading cause of chronic liver disease globally and can progress to cirrhosis, liver failure, and liver cancer. Current AASLD, AGA, and ADA guidelines recommend assessment for liver fibrosis in all patients with NAFLD. Serum biomarkers for fibrosis, while widely available, have notable limitations. Imaging-based noninvasive testing for liver fibrosis/cirrhosis is more accurate and is becoming more widespread.

**Methods:**

We evaluated the feasibility of a novel shear wave absolute vibroelastography (S-WAVE) modality called Velacur® for assessing liver stiffness measurement (LSM) for fibrosis and attenuation coefficient estimation (ACE) in differentiating patients with chronic liver disease from normal healthy controls.

**Results:**

Fifty-four healthy controls and 89 patients with NAFLD or cured HCV with a prior known LSM of >8 kPa were enrolled, and all subjects were evaluated with FibroScan® and Velacur®. Velacur® was able to discriminate patients with increased liver stiffness as determined by a FibroScan® score of >8 kPa from healthy controls with an AUC of 0.938 (0.88-0.96). For assessment of steatosis in NAFLD patients only, Velacur® could identify patients with steatosis from healthy controls with an AUC of 0.831 (0.777-0.880). The Velacur® scan quality assessment was superior in healthy controls, as compared to patients, and the scan quality, as assessed by the quality factor (QF) and interquartile range (IQR)/median, was affected by BMI. Velacur® was safe and well tolerated by patients, and there were no adverse events.

**Conclusion:**

Velacur® assessment of liver stiffness measurement and liver attenuation is comparable to results obtained by FibroScan® and is an alternative technology for monitoring liver fibrosis progression in patients with chronic liver disease. This trial is registered with NCT03957070.

## 1. Introduction

NAFLD is the most common cause of liver disease globally, and liver fibrosis is the prime determinant of liver-related morbidity and mortality [1, 2]. NAFLD is more prevalent in patients with type II diabetes mellitus and obesity, and a subset of patients with NAFLD are at risk of developing hepatic fibrosis, with progression to cirrhosis, HCC, liver failure, liver transplant, and death [3]. Liver biopsy remains the gold standard for the assessment of liver fibrosis/cirrhosis, but is an invasive procedure associated with morbidity, and is both time-consuming and resource-intensive [4]. Discordant biopsy results from the left and the right liver lobes, misclassification of cirrhosis possibly due to sampling, and significant interobserver and intraobserver variation in liver biopsy limits its use in assessment of liver fibrosis [5–7]. Liver stiffness, a surrogate for advanced fibrosis and cirrhosis as measured by transient elastography, is an independent predictor of liver failure, hepatocellular carcinoma (HCC), and mortality [8–11]. Multiple society guidelines advocate for noninvasive tests (NITs) for the assessment of fibrosis in patients with NAFLD or for those who are at risk of NAFLD based on the presence of diabetes and/or metabolic syndrome [12–14]. FIB-4, which uses aspartate aminotransferase, alanine aminotransferase, age, and platelets, has good diagnostic accuracy for advanced fibrosis in patients with NAFLD. It can be employed in an algorithmic assessment to rule out patients with advanced fibrosis with a negative predictive value of >90% at a cut-off score of <1.3. Advanced fibrosis in patients with NAFLD is suggested in patients with a FIB-4 score > 2.67, but it is estimated that 30-40% of patients assessed have values in the indeterminate range, and these two cohorts should be further assessed for fibrosis with elastography [14].

VCTE/FibroScan® is well-validated across a broad range of liver diseases and is the most commonly used ultrasound-based imaging modality for the assessment of liver fibrosis. FibroScan® has a limitation of a high failure rate of up to 27% of individuals with a high BMI using the standard M probe, likely due to the larger skin capsular distance (SCD) in obese individuals [15, 16]. Assessment of steatosis by CAP score and LSM is significantly affected by a SCD of >25 mm when using the FibroScan® M probe in patients with NAFLD [17]. The use of the obesity-specific (XL) probe has reduced the failure rate from 16% to 1.1% in individuals with BMI > 28 kg/m^2^ [18–20]. Other ultrasound technologies can assess LSM using point shear wave elastography (pSWE) and 2D shear wave elastography (SWE) methods which are currently commercially available on diagnostic ultrasound machines. Using the acoustic power of the ultrasound probe to create tissue displacement, the resulting wave is then tracked using ultrasound imaging techniques and liver stiffness is assessed [21].

These limitations can be overcome by using magnetic resonance elastography (MRE), which uses MR imaging to assess the shear waves within the liver tissue from an externally placed low-frequency vibration pad. MRE is less prone to operator error and sampling error, has a low technical failure rate, and as such is more accurate than FibroScan® [22, 23]. It has shown great reproducibility and low failure rate and is often effective in patients with high BMI. Success rates of 96% are recorded with MRE in patients with a BMI of >35 kg/m^2^ as compared to 88% in patients scanned with VCTE [24]. Studies demonstrate that MRE is more accurate than VCTE and pSWE in assessing patients with NAFLD and more accurate than VCTE in patients with chronic hepatitis [25, 26]. However, MRE is limited by its high cost, availability, and lack of scalability and may not be suitable for patients with claustrophobia which affects 0.7-2.1% of those scheduled [27].

Steatosis can be assessed by noninvasive imaging modalities. Ultrasound attenuation assesses the loss in signal power reported in decibels/minute (dB/m), and magnetic resonance imaging proton density fat fraction (MRI-PDFF) assesses the concentration of mobile triglycerides within tissue and can accurately assess hepatic steatosis. Ultrasound assessment of attenuation has shown a good correlation when compared to MRI-PDFF [28].

We propose a new method of shear wave absolute vibroelastography (S-WAVE), an ultrasound-based method using the medical device Velacur® (Sonic Incytes Medical Corp., BC, Canada) for the assessment of liver stiffness and liver steatosis and performed this prospective, open-label, feasibility, and clinical validation study of the Velacur® prototype system to differentiate between healthy volunteers and patients with liver fibrosis as assessed by FibroScan® measurements. This device is an ultrasound-based point-of-care technique which produces two- and three-dimensional images, is able to assess a large volume of hepatic tissue, allows for targeting of a specific region of interest, and is portable, allowing for scalability.

This prospective, open-label, feasibility, and clinical validation study of the Velacur® prototype system was performed at four liver care centers in Canada and the United States to evaluate the ability of Velacur® to differentiate between healthy volunteers and patients with liver fibrosis as assessed by FibroScan® measurements.

## 2. Methods

### 2.1. Device

**Figure 1:**
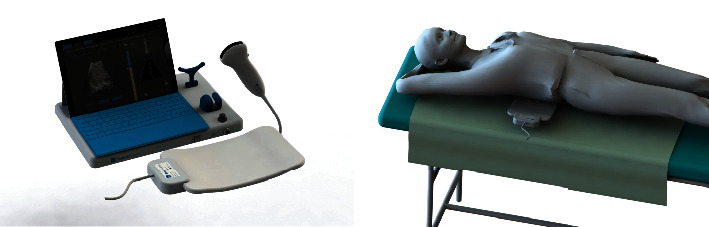
A prototype Velacur® system comprised of a control unit, a laptop, an ultrasound probe, and an activation unit.

For this study, a prototype Velacur® system was used. The device is comprised of a control unit, a laptop, an ultrasound probe, and an activation unit (Figure 1). The Velacur® system leverages the technological methods from both ultrasound and MRE. Velacur® measures liver stiffness through the creation and measurement of steady-state shear waves within the liver. Ultrasound attenuation is measured by the ultrasound beam strength.

### 2.2. Elasticity Assessment

**Figure 2:**
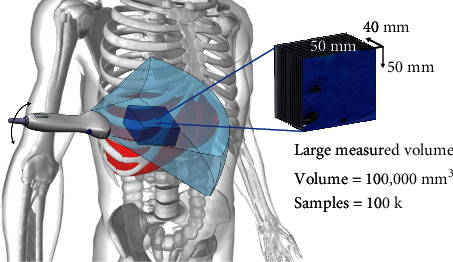
Velacur ultrasound probe is placed between the ribs to view the right lobe of the liver using an intercostal approach. A rendering of the general positioning of the probe, liver, and measured volume. The blue cone shows how much of the liver can be seen in the ultrasound image. The cube within the liver shows the large region that is used to measure the average elasticity and attenuation.

Steady-state shear waves are created within the patient's liver with the multifrequency (40-70 Hz) activation unit placed under the patient. The use of an external vibration source, similar to what is used in MRE, allows for multifrequency vibrations to produce shear waves deep into the liver of both smaller and larger BMI patients. Multiple frequencies of shear waves decrease the possibility of artifacts in the wave pattern affecting the final measurements and provide an opportunity for averaging thereby reducing measurement uncertainty. Elasticity results from all frequencies are averaged to create a result. A curvilinear abdominal ultrasound probe is used to image the right lobe of the liver using an intercostal approach allowing users to view the liver directly and ensure that measurements are made from liver tissue. Using a sweeping motion of the probe, facilitated by a sweep guidance tool, multiple planes of ultrasound data at a depth of 15 cm are acquired over 30 degrees, in the elevational direction. The sweep takes approximately 12 seconds during a breath hold to stabilize the liver position (Figure 2).

**Figure 3:**
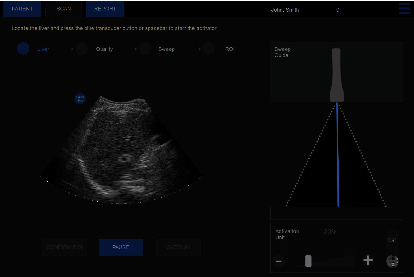
Velacur user interface showing the full B-mode image of the patient's liver and allowing the clinician to focus on a region of interest.

The shear wave field is measured over a volume (100 cm^3^) and used to produce the average volumetric spatial elasticity. Velacur® measures stiffness and attenuation in a volume of approximately 100 cm^3^ (Figure 2). At the end of the data collection, the user can define a region of interest (ROI) where measurements will be taken. The ability to view the liver on ultrasound allows for positioning of the ROI below the liver capsule, avoiding other artifacts and ensuring more accurate measurements (Figure 3). The algorithmic framework is based on previous work with updated algorithms for automatic shear wave data quality assessment and for automatic vessel detection within the liver [29, 30].

### 2.3. Attenuation Assessment

Attenuation measurements are made from the ultrasound data captured simultaneously with elasticity measurements. The attenuation is computed by measuring the drop in the power of the ultrasound signal with depth as compared to the drop in power measured using a reference phantom with a known ultrasound attenuation [31]. Attenuation results from Velacur® are expressed as the attenuation coefficient estimation (ACE).

### 2.4. Reporting

A minimum of 10 measurements on all subjects are performed to assess liver elasticity and ACE for the purposes of this study. At the end of each sweep, a quality measure (expressed as a percentage), elasticity (in kPa), and attenuation (dB/m) are recorded. The final result is calculated as the median of the collected measurements. The interquartile ranges for both elasticity and attenuation are displayed as a further indication of the exam quality.

### 2.5. Study Participants and Design

The study was a prospective, open-label, feasibility, and clinical validation study of the Velacur® prototype system including patients with liver fibrosis (cohort 1, *N* = 86) and healthy volunteers (cohort 2, *N* = 54) for a total of 140 participants. This multicenter study enrolled patients at four sites in Canada and the United States: LAIR Center (Vancouver, BC, Canada), Vancouver Coastal Health (Vancouver, BC, Canada), Beth Israel Deaconess Medical Center (Boston, MA, USA), and Southern California Research Center (Coronado, CA, USA). This study represents the first clinical study using the S-WAVE technology in patients with chronic liver disease.

The study was completed in accordance with Good Clinical Practices and received IRB or REB approval for enrollment at each site with the following protocol numbers: WIRB 20182643, VCH H19-01178, BIDMC 2018P000730, and WIRB 20182643. All study participants provided written informed consent prior to inclusion in the study.

### 2.6. Study Objectives

The purpose of this study was to establish the discriminatory ability of Velacur® to differentiate healthy volunteers from patients based on liver elasticity and ultrasound attenuation levels. The patient's disease state was based on FibroScan® results and on the patient's disease history.

The safety and tolerability of Velacur® were assessed by measuring the number of reported adverse events and any studies discontinued at the request of the subject.

The feasibility of using the Velacur® prototype system for liver elasticity and attenuation measurements was evaluated.

### 2.7. Inclusion and Exclusion Criteria

Male and female patients between the ages of 19 and 75 with known a clinical diagnosis of NAFLD or with cured (SVR 12 or 24) chronic hepatitis C virus (HCV) infection and with a historical FibroScan of between 8 and 35 in the past 13 months were recruited. A prior diagnosis of NAFLD was defined by one of the following within 12 months: biopsy-proven NAFLD/NASH, a previous MRI-PDFF greater than 12%, abdominal ultrasound with evidence of hepatic steatosis, and FibroScan® CAP score greater than 230 dB/m. Patients with at least 2 criteria for metabolic syndrome and increased liver stiffness were also included.

A control group of healthy volunteers between 19 and 75 years old, with no history of liver disease or alcohol use disorder, were enrolled.

Patients and healthy subjects were excluded if they had current HCV infection, other liver disease diagnoses, known coinfection with other hepatitis viruses or human immunodeficiency virus (HIV), decompensated cirrhosis, or persistent alcohol consumption exceeding 20 g of alcohol per day. Individuals with implanted electrical devices such as pacemakers, internal defibrillators, cochlear implants, and nerve stimulators and pregnant people were excluded. Patients with a body mass index (BMI) of ≥40 kg/m^2^ were excluded due to the known failure of FibroScan® at higher body mass index (BMI).

Patient disease state, height, weight, gender, age, race, and prior treatment history were recorded. Historical ALT, AST, and platelet counts were collected for patients. All participants were required to be fasting for at least 3 hours before the beginning of either FibroScan® or Velacur® exams which were performed on the same day unless specific conditions prevented it.

FibroScan® was performed on all participants by a trained and experienced FibroScan® user in accordance with the FibroScan® instructions for use. At least 10 valid scans were collected, using the M or XL probes as recommended by the Echosens algorithm. The median value of the 10 measurements was used as the final result, and the IQR/median was recorded.

Velacur® scans were performed on all participants by users who were trained, but otherwise inexperienced with Velacur®. All users went through the same two half-day hands-on training program with a Sonic Incytes trainer before the initiation of the trial. A total of ten sweeps were collected for each participant. The median of the 10 measurements was used as the final result, and the IQR/median was recorded.

For each Velacur® measurement, a quality factor (QF) measure is recorded. This software-derived QF is a Velacur® feature, which is a combination of B-mode image quality, tissue displacement, user motion during data collection based on the vibration, and the ability of the user to complete a smooth and regular sweep motion during image acquisition. This is an indication that a successful scan was completed. A QF above 60% is considered to be a valid sweep. The ACE (attenuation coefficient estimate) quality factor is derived only from the attenuation measurements.

## 3. Statistical Analysis Plan

### 3.1. Discriminatory Ability

The discriminatory ability of Velacur® to differentiate liver stiffness and ultrasound attenuation of healthy volunteers from the patients was assessed. An AUROC and 95% confidence intervals (CI) using final Velacur® elasticity measurement as a predictor of healthy versus fibrotic liver was constructed. Ground truth was established separately using both the patient's disease status (history or no history of liver disease) and the FibroScan® results on the day of the scan.

### 3.2. Safety and Tolerability

The description and frequency of adverse events related to Velacur® experienced by the patient or healthy subjects were collected.

### 3.3. Feasibility

The QF for all scans is summarized by the proportion of all scans that are deemed satisfactory. FibroScan® uses the interquartile range (IQR) divided by the median measurement as a tool to quantify exam quality. The association between IQR/median and participant characteristics are evaluated using Wilcoxon-Mann-Whitney for continuous variables and chi-squared tests for discrete variables. The associations are evaluated with IQR/median for Velacur® and FibroScan®.

## 4. Results

Fifty-four health controls and 86 patients (59 NAFLD and 27 cured HCV) were included in the study. Healthy controls were recruited based on clinical history completed via interviews. Of the 140 enrolled subjects, 133 of them have complete data sets and were included in the analysis, for a final number of 54 healthy, 54 NAFLD, and 25 HCV SVR participants. Seven patients (5%) were excluded from the analysis due to technical factors. The factors were related to the Velacur® device and included cable or computer memory malfunctions in 6 cases, and in 1 case, the device could not be powered due to infrastructure issues. All device-related issues occurred early in the study and were quickly rectified.

Healthy controls were significantly younger than patients (mean age 28 versus 59 years; *P* < 0.001) (Table 1). Median liver stiffness as assessed by Velacur® and FibroScan® was expectedly higher in patients than healthy controls without liver disease (Table 2). Similarly, attenuation measurements for hepatic steatosis were also significantly higher in the patients than in the healthy controls using both Velacur® and FibroScan® (Table 2).

### 4.1. Discriminatory Ability of Velacur for Patients and Healthy Volunteers

Velacur® was able to discriminate patients with increased liver stiffness as determined by a FibroScan® score of >8 kPa from healthy controls with an AUC of 0.938 (95% CI 0.88-0.96) (Table 3). Additionally, Velacur® could discriminate NAFLD patients from healthy controls with an AUC of 0.9280 (95% CI 0.8637-0.9636) (Table 3). Steatosis cutoffs for FibroScan® used for the determination of steatosis were derived from a study by Sasso et al. [32]. Fourteen of the volunteers had CAP scores > 238 dB/m on FibroScan®, possibly indicating the presence of steatosis. Four patients with NAFLD had CAP scores of <238 dB/m. When the analysis was completed on all subjects using CAP as the ground truth, separating subjects based solely on their CAP score, the AUC calculated for Velacur attenuation is calculated to be 0.8458 (0.775-0.915). This indicates that Velacur ACE may be better able to separate patients with NAFLD than the current CAP cut-off of 238 dB/m.

### 4.2. Safety and Tolerability

Velacur® was safe and well tolerated by patients; no patients requested to end the exam before it was complete. Overall, there were no adverse events related to the use of the device and no adverse events which required any follow-up with the patient.

### 4.3. Feasibility of Velacur®

A total of 10 users were trained across 4 sites for this study. All users were either research assistants or clinical trial coordinators and were trained to use Velacur® just prior to study initiation. Only one user has previous experience with abdominal ultrasound.

In 123/133 (91%) of cases, users were able to achieve the 60% quality factor for the Velacur® scan. The average BMI for these 10 patients that were excluded was 33.3 kg/m^2^, higher than the average BMI for the entire cohort; all patients were recruited from a single site early in the trial and it is likely that higher BMI and inexperience users resulted in difficulty obtaining scan results and contributing to the low quality for these patients.

A lower IQR/median is an indication of a more consistent scan. It is recommended that an IQR/median of 30% be used as an indication of a reliable scan for both stiffness and attenuation measurements. There was a significant difference shown between the ACE IQR/median of healthy volunteers and patients, but not in the Velacur® elasticity (Table 4). This shows that the Velacur stiffness measurements were consistent across all disease states evaluated. Significant associations were also shown between the Velacur® elasticity IQR/median and the BMI of all participants. Using the IQR/median, lower ACE IQR/median was found in healthy volunteers reflecting better scan quality. In 86/133 (63%) cases, the Velacur® elasticity IQR/median was less than 35%. In 128/133 (96%) of the subjects, the ACE IQR/median was less than 30%. This shows that the majority of scans were of high quality.

For FibroScan® measurements, there were significant associations between the IQR/median for CAP measurements and all patient characteristics. For the elasticity measurements, there were no significant differences between the IQR for the healthy volunteer cohort and the patients (Table 4).

In 132/133 (99%) cases, the FibroScan® elasticity IQR/median was less than 35%, while for 120/133 (90%) of the CAP, the IQR/median was less than 30%.

## 5. Discussion

NAFLD is the most common cause of liver disease globally, and increasing liver stiffness as assessed by transient elastography is an independent risk factor for liver-related mortality. Societal guidelines advocate for the assessment of fibrosis in patients with NAFLD or those who are at risk of NAFLD using NITs and include transient elastography as a tool for assessing liver fibrosis. Current NITs have well-recognized limitations or accuracy and scalability in this population of patients. Additionally, most patients with NAFLD or NASH are seen in primary care or endocrine clinics that are ill-equipped to assess for progressive liver disease.

In this pilot study, we assess the feasibility of a novel Velacur® prototype in a population of healthy volunteers and patients with increased levels of fibrosis. We also looked at the ability of Velacur® to discriminate between patients with chronic liver disease and healthy volunteers with no known liver disease history. Velacur® was able to discriminate patients with liver fibrosis as assessed by FibroScan® from healthy controls without known liver disease or liver fibrosis with an AUROC of 0.938 (95% CI 0.88-0.96) which is considered excellent as a performance metric. Velacur® has excellent discriminate abilities to determine the presence of steatosis in a cohort of NAFLD patients as compared to healthy controls with an AUC of 0.928 (95% CI 0.864-0.964).

Velacur was well tolerated by all patients. No patients requested to end the exam before completion. Some did have trouble with the 12-second breath hold, and improvements are underway to decrease the breath hold requirements and improvements in technology decreased the breath hold to 8 seconds on more recent devices.

As a novel and investigational device, users were inexperienced at the onset of the study. Most had very limited previous experience with only 1 operator having any experience with standard ultrasound technique. As noted above, there were some technical difficulties which resulted in the exclusion of some enrolled patients, mostly due to incomplete scans.

When looking at the IQR/median as a measure of the exam quality, the IQR could be heavily influenced by the BMI of the participants, as the BMI of patients was significantly higher than that of the healthy volunteers. Velacur® shows some dependence of elasticity IQR on the patient's BMI, but there was no difference in the ACE IQR with BMI, showing that the fat measurement is not affected by higher skin-to-capsule distance. FibroScan® CAP did show differences in CAP IQR for all patient characteristics, indicating that the CAP reliability may be influenced by the patient body type and other factors.

We recognize that our study has some limitations. As a feasibility study, we used a FibroScan® cut-off of >8 kPa to recruit people with moderate to advanced fibrosis. It is known that FibroScan® is not the usual gold standard for assessment, but as the most commonly used device in clinical practice to measure liver stiffness and ultrasound attenuation, it was chosen as the comparator for this feasibility study. Additionally, since the advent of NITs for liver fibrosis assessment, the frequency of liver biopsy for liver fibrosis assessment outside of clinical trials is declining significantly. In a recent survey, only 24% of gastroenterologists and hepatologists routinely perform liver biopsy in patients with presumed NAFLD [33]. It is possible that some of the patients or healthy volunteers were misclassified in the course of this comparison. During the study, some of the healthy volunteers were found to have elevated FibroScan® CAP scores and elevated Velacur® ACE scores. All volunteers were enrolled based on history, and there were no other tests completed to further validate this assumption. Recent studies of the prevalence of fatty liver in young adults in the general population have shown that up to 20% have hepatic steatosis, and so it is not unexpected that some healthy volunteers will have elevated attenuation scores [34]. After excluding these patients, the ability to detect steatosis was not unduly affected. In order to improve the accuracy of fibrosis and steatosis assessment in future studies, we are using MRE and MRI-PDFF to assess for fibrosis and steatosis.

## 6. Conclusions

In conclusion, this study demonstrates the feasibility of using Velacur® in a point-of-care setting, with its successful use in four clinics in Canada and the United States associated with no device-related adverse events. Velacur® was successfully able to differentiate healthy volunteers from patients with moderate or advanced liver stiffness according to FibroScan® and medical history and differentiate those with normal and higher levels of ultrasound attenuation.

## Figures and Tables

**Table 1:** Patient and health control characteristics.

	Healthy	Patients	*P* value (where applicable)
Number of enrolments	54	86 (59 NAFLD, 27 HCV SVR)	
Average age (range)	28 (19-61)	59 (31-75)	<0.0001
Average BMI (range)	23.9 (17-31.7)	30.9 (18.8–41.6)	<0.0001
Gender	16/54 (30%) male	54/85 (63%) male	<0.0001
Median AST	NA	28 (6–367)	NA
Median ALT	NA	30 (9–217)	NA
Median platelets	NA	201.5 (63-536)	NA
Median FIB-4	NA	1.475 (0.33–7.165)	NA

**Table 2:** Velacur™ and FibroScan® assessments of LSM and attenuation parameters in healthy controls and patients.

	Healthy	Patients (all)	*P* value (where applicable)	Patients (NAFLD)	Patients (HCV SVR)	*P* value (where applicable)
*N*	54	79		54	25	
Velacur™ stiffness	6.656 ± 0.82	7.67 ± 1.67	<0.0001.	7.18 ± 1.61	8.0 ± 1.69	0.036
FibroScan® stiffness	4.64 ± 1.35	12.76 ± 5.62	<0.0001	12.16 ± 5.55	13.53 ± 5.75	0.202
FibroScan® probe used	M = 50 (92%), XL = 4	M = 37 (47%), XL = 42	<0.0001	M = 20 (37%), XL = 34	M = 17 (72%), XL = 8	0.0025
Velacur™ ACE	215.99 ± 42.05	279.73 ± 60.67	<0.0001	313.73 ± 57.41	286.63 ± 64.53	0.084
FibroScan® CAP	207.31 ± 45.72	293.65 ± 65.35	<0.0001	320.15 ± 47.53	249.79 ± 73.2	<0.0001

**Table 3:** AUROC for Velacur™ assessments of LSM and attenuation parameters in different subject cohorts.

	Number of subjects	AUROC Velacur E (95% CI)	AUROC Velacur ACE (95% CI)
Healthy controls vs. patients	54 vs. 79	0.9379 (0.8805-0.9636)	0.9011 (0.8451-0.9454)
Healthy control vs. NAFLD patients	54 vs. 54	0.9156 (0.8617-0.9592)	0.9280 (0.8637-0.9636)
Healthy control vs. HCV SVR patients	54 vs. 25	0.9859 (0.9492-0.9969)	0.8430 (0.6831-0.9137)
Using FibroScan < 8 kPa and >8 kPa	62 vs. 71	0.9137 (0.8648-0.9564)	N/A
Using CAP < 238 dB/m and >238 dB/m	62 vs. 71	N/A	0.8458 (0.7753-0.9150)

**(a):** 

IQR/median	Statistical test	Velacur elasticity IQR/median				Velacur ACE IQR/median			
		Mean IQR/median (%)		*P* value	Sig	Mean IQR/median (%)		*P* value	Sig
Healthy vs. patient	Rank sum	Healthy (34.20 ± 17.56)	Patient (29.14 ± 16.85)	0.13	No	Healthy (13.08 ± 7.03)	Patient (10.91 ± 7.54)	0.03	Yes
Gender	Rank sum	Male (32.56 ± 18.58)	Female (29.91 ± 15.92)	0.10	No	Male (10.48 ± 5.98)	Female (13.04 ± 8.37)	0.10	No

**(b):** 

IQR/Median	Statistical test	FibroScan elasticity IQR/median				CAP IQR/median			
		Mean IQR/median (%)		*P*	Sig	Mean IQR/median (%)		*P*	Sig
Healthy vs. patient	Rank sum	Healthy (13.58 ± 6.71)	Patient (14.45 ± 7.86)	0.13	No	Healthy (24.33 ± 17.4)	Patient (12.9 ± 9.99)	0.03	Yes
Gender	Rank sum	Male (13.69 ± 7.33)	Female (14.48 ± 7.50)	0.10	No	Male (14.63 ± 10.35)	Female (20.42 ± 17.29)	0.10	No

**(c):** 

IQR/median	Statistical test	Velacur				FibroScan			
		Elasticity IQR/median		ACE IQR/median		Elasticity IQR/median		CAP IQR/median	
		*P*	Sig	*P*	Sig	*P*	Sig	*P*	Sig
BMI	Spearman correlation	0.0017	Yes	0.18	No	0.15	No	<0.001	Yes
Age	Spearman correlation	0.085	No	0.33	No	0.37	No	<0.001	Yes

## Data Availability

The data from which the results of this study were obtained is available upon request from Caitlin Schneider, Sonic Incytes Medical Corp., Vancouver, BC, Canada, caitlin@sonicincytes.com.

## Abbreviations

NAFLD: Nonalcoholic fatty liver disease
S-WAVE: Shear wave absolute vibroelastography
LSM: Liver stiffness measurement
NASH: Nonalcoholic steatohepatitis
AUC: Area under the curve
kPa: Kilo Pascal
QF: Quality factor
IQR: Interquartile range
BMI: Body mass index
NITs: Noninvasive tests
MR: Magnetic resonance
MRE: Magnetic resonance elastography
Hz: Hertz.
